# A biomechanical assessment of modular and monoblock revision hip implants using FE analysis and strain gage measurements

**DOI:** 10.1186/1749-799X-5-34

**Published:** 2010-05-12

**Authors:** Habiba Bougherara, Rad Zdero, Suraj Shah, Milan Miric, Marcello Papini, Paul Zalzal, Emil H Schemitsch

**Affiliations:** 1 Department of Mechanical and Industrial Engineering, Ryerson University, Toronto, ON, M5B-2K3, Canada; 2 Martin Orthopaedic Biomechanics Laboratory, St. Michael's Hospital, Toronto, ON, M5B-1W8, Canada; 3 Department of Surgery, McMaster University, Hamilton, ON, L8S-4L8, Canada; 4 Department of Surgery, University of Toronto, Toronto, ON, M5G-1L5, Canada

## Abstract

**Background:**

The bone loss associated with revision surgery or pathology has been the impetus for developing modular revision total hip prostheses. Few studies have assessed these modular implants quantitatively from a mechanical standpoint.

**Methods:**

Three-dimensional finite element (FE) models were developed to mimic a hip implant alone (Construct A) and a hip implant-femur configuration (Construct B). Bonded contact was assumed for all interfaces to simulate long-term bony ongrowth and stability. The hip implants modeled were a Modular stem having two interlocking parts (Zimmer Modular Revision Hip System, Zimmer, Warsaw, IN, USA) and a Monoblock stem made from a single piece of material (Stryker Restoration HA Hip System, Stryker, Mahwah, NJ, USA). Axial loads of 700 and 2000 N were applied to Construct A and 2000 N to Construct B models. Stiffness, strain, and stress were computed. Mechanical tests using axial loads were used for Construct A to validate the FE model. Strain gages were placed along the medial and lateral side of the hip implants at 8 locations to measure axial strain distribution.

**Results:**

There was approximately a 3% average difference between FE and experimental strains for Construct A at all locations for the Modular implant and in the proximal region for the Monoblock implant. FE results for Construct B showed that both implants carried the majority (Modular, 76%; Monoblock, 66%) of the 2000 N load relative to the femur. FE analysis and experiments demonstrated that the Modular implant was 3 to 4.5 times mechanically stiffer than the Monoblock due primarily to geometric differences.

**Conclusions:**

This study provides mechanical characteristics of revision hip implants at sub-clinical axial loads as an initial predictor of potential failure.

## Background

About 150,000 total hip arthroplasty (THA) surgeries are performed each year in the United States [1]. This is the most common surgical treatment for hip osteoarthritis and rheumatoid arthritis, which may begin as a complex mechano-chemical degenerative cascade [2]. Subsequent revision hip arthroplasty may be necessitated by component dislocation, protrusion, malalignment, wear, fracture, loosening, sepsis, and/or osteolysis of the original implant [3].

During revision hip surgery, the primary hip implant is removed, and the femoral canal is reamed to a deeper point to receive the longer stems of a revision hip implant, which sometimes compensates successfully for bone loss due to surgery, trauma, or pathology. Bone grafts may also be used to augment bone during revision. The aim of revision surgery is to relieve pain and restore proper hip function. A challenge faced by surgeons in revision arthroplasty is adequate fixation of the new implant because of the loss of femoral bone stock incurred due to initial trauma, primary hip implant surgery, or pathology.

Revision hip stems are usually constructed of a single piece of titanium or cobalt-chrome alloy onto which a spherical femoral head is subsequently mounted. New modular stem implants have been recently developed consisting of perfectly interlocking proximal and distal components [4-9]. The Zimmer Modular Revision Hip System (Zimmer, Warsaw, IN, USA) has been assessed clinically and radiographically with good results with respect to pain, stiffness, function, and subsidence, with few complications related to dislocation, infection, and intra-operative femur fracture, albeit for short follow-up times of 2 to 5 years [10,11]. No study to date has quantitatively assessed this implant for mechanical behavior.

The present aim was to compare the mechanical characteristics of two revision hip stems, namely, an interlocking two-piece Modular hip implant versus a traditional-type single-piece Monoblock hip implant. A finite element (FE) model of both hip stems was developed to assess strain and stress distribution under sub-clinical static axial loads and compared to mechanical tests. Present results may provide preliminary information to clinicians in deciding whether modular or single-piece devices are preferred for a specific patient group.

## Methods

### Modular and Monoblock hip implants

**Figure 1:**
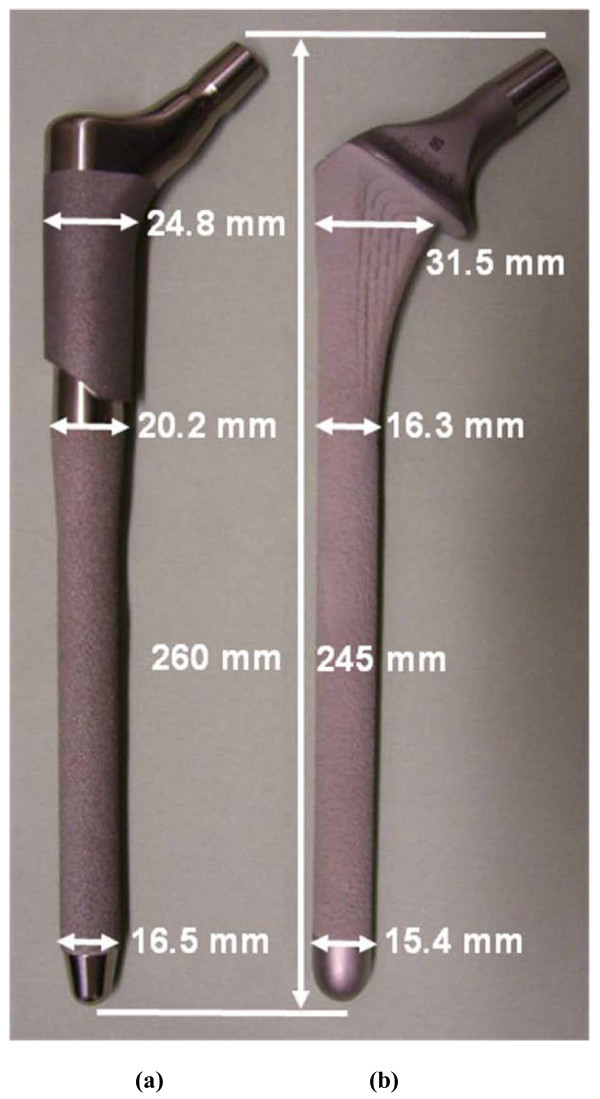
**Hip implants used in this study**. (a) two-piece Modular and (b) single-piece Monoblock. Dimensions were measured by the authors and may differ slightly from those provided by the manufacturer. Photographs are not to scale relative to one another.

The Modular interlocking two-piece stem (Zimmer Modular Revision Hip System, Zimmer, Warsaw, IN, USA) had a proximal "taper" body and distal "porous" stem, as termed by the manufacturer (Figure 1a)[12]. Attachment of the body to the stem was accomplished by a compression nut placed over the threaded stem tip torqued to 15 Nm. For the stem, the proximal smooth surface was treated with a hardening process, while the distal surface was roughened to maintain Ti-6Al-4V substrate strength and allow for bone ingrowth. The stem had a total length of 260 mm and a medio-lateral width ranging from 16.5 mm to 24.8 mm. The Monoblock single-piece stem (Stryker Restoration HA Hip System, Stryker, Mahwah, NJ, USA) was manufactured from a single piece of Ti-6Al-4V (Figure 1b)[13]. The implant had a calcar collar. To enhance bone ingrowth, the surface was roughened and coated with hydroxylapatite (HA). The stem had a total length of 245 mm and a medio-lateral width ranging from 15.4 mm to 31.5 mm. Both implants are meant to be used in a cementless manner.

### Finite element (FE) models

#### General approach

**Figure 2:**
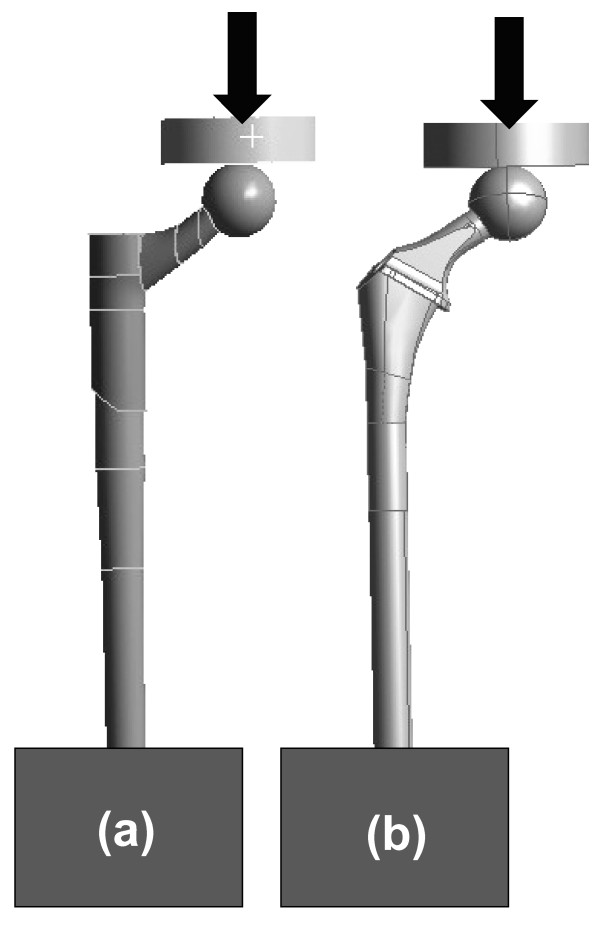
**CAD models for hip implants alone (Construct A)**. (a) Modular and (b) Monoblock devices were each placed in rigid blocks distally and then proximally subjected to 700 and 2000 N axial loads using a flat plate during FE analysis. The same flat plate was used in applying load in FE analysis for the hip implant-femur (Construct B). Large arrows show vertical load direction.

**Figure 3:**
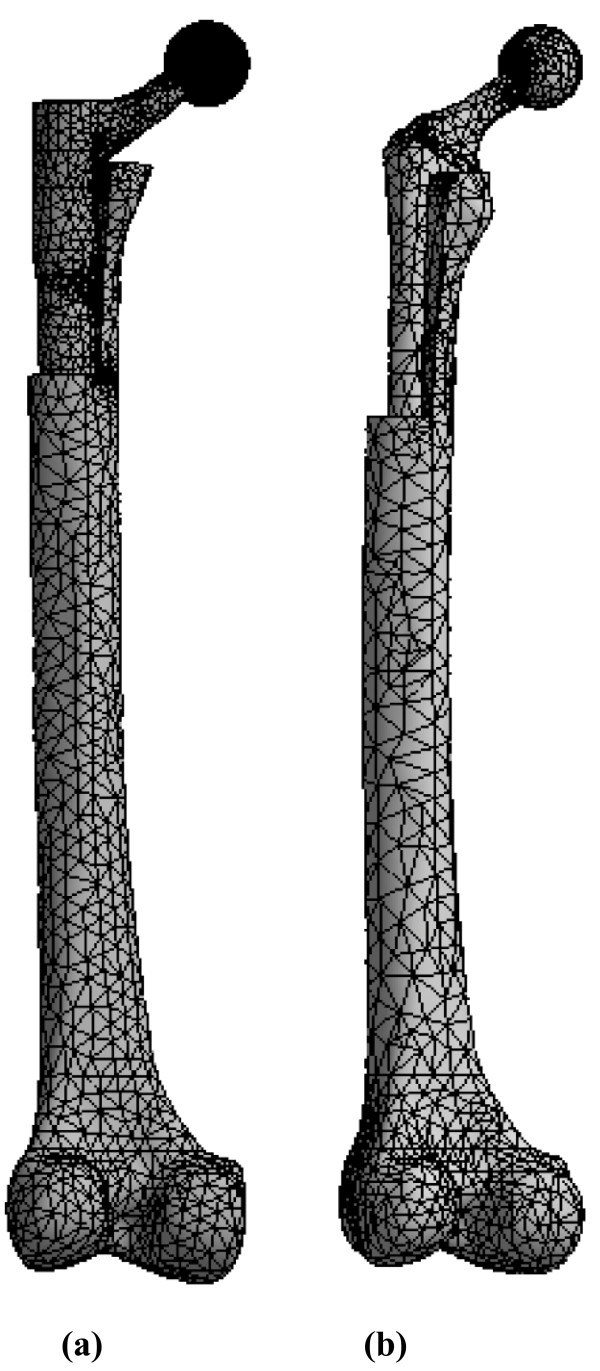
**FE mesh models for hip implant-femur (Construct B)**. (a) Modular and (b) Monoblock devices. Femurs had a proximal step-like cut to simulate bone loss from trauma, pathology, and/or surgical shaping. Vertical force applicator and rigid distal fixation are not shown. Posterior view is shown.

FE models of the Modular and Monoblock implants were developed, and strain and stress maps were generated for two constructs, A and B. Construct A modeled a hip implant alone under axial forces of 700 and 2000 N (Figure 2). Construct B modeled a hip implant-femur (Figure 3) under an axial force of 2000 N. Construct A FE data for Modular and Monoblock devices were validated experimentally at 700 and 2000 N to endorse the results of the implants themselves used in the FE model of Construct B. The FE model of the synthetic intact femur was validated experimentally by the current authors for axial and torsional stiffness [14,15] and while instrumented for surface strain [15,16].

#### CAD model of hip implants

SolidWorks 2007 CAD software (SolidWorks Corp., Dassault Systèmes Concord, MA, USA) was used to create a solid model of the Modular and Monoblock hip implants. Models of the implant system were created in SolidWorks. All models of the implant were generated based on the geometries of specimens used in the experimental trials (see Experimental Methodology below).

#### CAD model of synthetic femur

The CAD model of the large left Third Generation Composite Femur (Model No. 3306, Pacific Research Labs, Vashon, WA, USA) was developed earlier [14-16]. Computerized tomography (CT) scans were performed every 0.5 mm along the synthetic femur and exported in SolidWorks CAD software. The CAD model contained surfaces representing cortical and cancellous bone, but had no intramedullary canal. The canal was created by cutting away cancellous bone. The femur had a proximal step-like section removed of 95 mm distal-proximal length and 16 mm medial-lateral width. This simulated traumatic, pathological, or surgically-generated bone loss during or following total hip replacement surgery in scenarios where there may be no supportive proximal femoral bone and the diaphyseal structural support is compromised or minimal. This may be augmented by a bone graft in order to restore the greater trochanter and/or abductor muscle attachment.

Although the FE model of the femur could have been based on CT scans of a human femur, the authors chose to use a synthetic femur for the following reasons, namely, the synthetic femur's mechanical properties have been validated against human femurs previously, the FE model of the synthetic bone was already available to the authors, the inter-specimen uniformity of the synthetic femurs would allow for ease-of-comparison to future FE and experimental studies using this often-used commercially-available synthetic bone, and the ease-of-storage and lack of degradation of the synthetic bone permitted the authors to frequently re-examine the bone itself during the study, unlike cadaveric material.

Prior experimental validation of the same FE model of the femur itself showed only moderate average differences between experimental and FE values for axial stiffness (0% difference; cortical bone E = 9.1 GPa; intact femur)[14], torsional stiffness (3.3% difference; cortical bone E = 9.1 GPa; intact femur)[14], medial surface strain (10% difference; cortical bone E = 10 GPa; intact femur)[15], and medial surface strain (10.9% difference; cortical bone E = 10 GPa; instrumented femur)[16].

The same geometry and material properties for the femur were used in previous works [15,16]. In addition, the same tetrahedral elements of identical shape and size were employed to mesh the femur. Mesh sensitivity was done on the model using a Workbench mesh tool called "relevance", which indicates the minimum number of elements (coarse mesh, 0% relevance) to maximum number of elements (very fine mesh, 100% relevance) possible to discretize the femur. A preliminary investigation showed that an 80% mesh relevance model of the femur had stress and strain values less than 1% different from a 100% mesh relevance model of the femur. Thus, an FE femur model with 80% relevance was used presently. The number of total elements used, of course, was different from previous works because the present model required removing proximal bone to accommodate the total hip stems.

#### Femur-Implant Assembly

The femur-implant systems (Construct B) were created in SolidWorks by assembling the individual models of the femur, the implant, the cement square fixation block, and the flat plate load applicator. The assembly was exported to ANSYS Workbench 11.0 for FE analysis. The WorkBench "simulation" module automatically generates contact between the assembled surfaces. CONTA174 in ANSYS is a three-dimensional 8-node surface-to-surface contact element that was used in this study. This type of contact element was located on a deformable surface of a three-dimensional solid element that contacts and slides on a target surface, i.e., TARGE170 in this study. CONTA174 had three degrees of freedom at each node, namely, translations in the nodal x, y, and z directions. It had the same geometric characteristics as the solid element face with which it was connected. Contact occurred when the element surface penetrated its associated target element, i.e., TARGE170. CONTA174 and TARGE170 shared the same real constants. All contact elements were set to fully bonded, which in reality is equivalent to a coefficient of friction of 1. Bonded contact was used to simulate full bony ongrowth and long-term mechanical stability.

The FE model of the flat plate load applicator was created using a 20-node structural solid containing 52791 nodes and 11924 elements (Figure 2). It was assigned material properties of steel (E = 200 GPa, ν = 0.3). The flat plate had a 20 mm thickness and a 70 mm diameter. A force defined by a vector was applied on the top surface of the flat plate, resulting in a 700 or 2000 N axial load. These loads acted to apply a vertical force onto the metallic femoral ball of both hip implants. The movement of the flat plate was restricted in all directions, except in the vertical direction. This arrangement was used for both Constructs A and B.

#### Meshing and material properties

ANSYS Workbench 10.0 was used to generate meshes. For Construct A, the number of nodes and elements was 41098 and 29436 (Modular) and 33270 and 23744 (Monoblock). For Construct B, the number of nodes and elements was 52121 and 39423 (Modular) and 61577 and 45847 (Monoblock). Body elements included 10-node quadratic tetrahedrons for cortical bone, cancellous bone, and implants, and a 20-node quadratic hexahedron for the axial force applicator. Contact elements were quadratic triangular for cortical bone, cancellous bone, and implants, and quadratic quadrilateral for the axial force applicator. Synthetic femurs were isotropic and linearly elastic, with material properties for cortical (E = 10 GPa, ν = 0.3) and cancellous (E = 206 MPa, ν = 0.3) bone based on prior studies [14-16]. Young's Modulus for cortical bone (E = 10 GPa) was an average of compressive (7.6 GPa) and tensile (12.4 GPa) values [15]. The Modular stem is made from a titanium-based substrate that has been surface hardened but whose properties are proprietary information (U.S. Patents 5,192,323 and 5,326,362) and Ti-6Al-4V, a well-known industrially used titanium-based product. The Monoblock stem, however, is manufactured solely from Ti-6Al-4V. Titanium-based alloys have a typical Young's modulus range of 100 to 120 GPa. Thus, material properties for both of these titanium-based implants were set in the middle of the range for titanium alloy (E = 110 GPa, ν = 0.36). The femoral balls were set for cobalt-chrome (E = 200 GPa, ν = 0.3).

#### FE analysis

FE analysis was done using ANSYS Workbench 10 suite to replicate experimental conditions. For Construct A, experimental cement potting of the hip stems was mimicked in the Simulation utility by constraining the distal 25 mm of the stem lengths. For Construct B, the displacement of the distal end of the femur was restrained by assigning displacement restrictions on the distal faces of the femur. Vertical forces were applied at the face of the applicator with motion restricted in all but the axial direction (i.e., z-axis). Bonded contact was modeled between all contact surfaces, namely, bone-implant, implant-cement, and implant-implant interfaces. Also, the contact region between the vertical load applicator and cobalt-chrome femoral ball was bonded with no slipping. The FE models for Construct B (hip stems implanted into femurs) mimicked the long-term stability of the implants. The bone-implant interfaces, modeled as fully bonded, would be representative of the bony ongrowth around the hip stems that would be expected to occur over the long-term.

### Experimental Methodology

#### General test parameters

**Figure 4:**
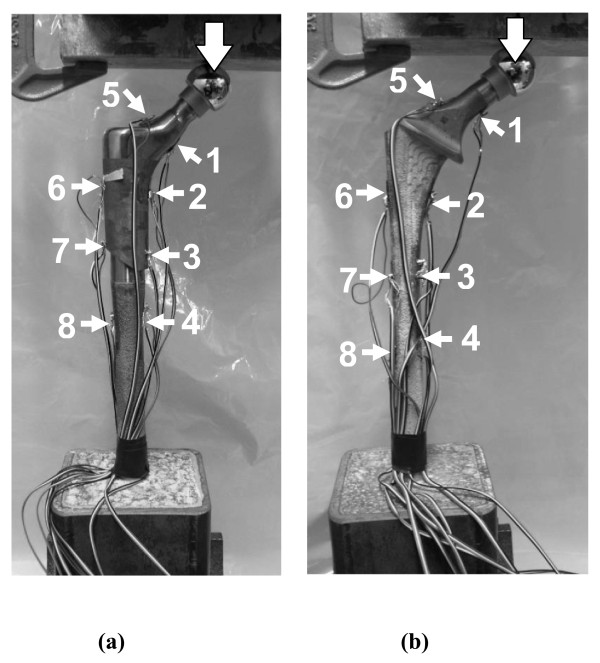
**Mechanical testing of hip implants alone (Construct A)**. (a) Modular and (b) Monoblock devices. An indenting plate compressed the femoral head. Implants were fixed in cement chambers to a depth such that working lengths for both stems were 225 mm. Large arrows show vertical load direction. Strain gages are indicated by numbered arrows.

The FE model of Construct A was validated with mechanical tests at 700 and 2000 N of axial load (Figure 4). Axial load levels were similar to those previously used for intact femurs and hip implant-femur constructs [14-22].

#### Hip implant preparation

Distal ends of the hip prostheses were placed into square steel chambers filled with cement. Implants were positioned vertically and inserted so the working lengths were 225 mm. Implants were instrumented with 350 Ohm linear strain gages (Model CEA-06-125UW-350, Vishay Measurements Group, Raleigh, NC, USA). Each prosthesis had 8 gages fixed along medial and lateral surfaces. Because of differing geometries and surface texturing of the implants, strain gage locations could only be placed approximately at corresponding points. Wire leads from each gage were attached to an 8-channel Cronos-PL data acquisition system (IMC Mess-Systeme GmbH, Berlin, Germany). FAMOS V5.0 software (IMC Mess-Systeme GmbH, Berlin, Germany) was used for data storage and analysis.

#### Mechanical testing

Experiments were performed using an Instron 8874 (Canton, MA, USA) with a capacity of ± 25 kN, a resolution of 0.1 N, and an accuracy of ± 0.5%. Hip implants had a cobalt-chrome femoral ball (diameter, 26 mm) and were distally secured with a vice. A vertical load was applied using a flat metal plate under displacement control (displacement ≤ 0.5 mm; rate = 5 mm/min; preload = 50 N). The slope of the "ramp-up" force-displacement curve was the axial stiffness. This was repeated three times, and an average value was computed.

#### Strain gage measurement

A second series of tests on each implant achieved 700 and 2000 N axial loads. Based on initial implant stiffness obtained earlier, a maximum deflection was computed and used to reach the desired axial force. Strain values were recorded for a minimum of 60 seconds and averaged with little variation during this period. Tests were done with the same preload and loading rate described earlier and were repeated three times at 700 and 2000 N axial forces to obtain average strain for each gage. Load control, though usually used to achieve a fixed force, was unstable at present; thus, all experiments were done with displacement control.

### Percentage Difference Calculations

For the implant alone (Construct A), the absolute values of the differences between FE strains and experimental measurements were calculated as % difference = (FE strain - experimental strain)/experimental strain × 100. For the implant-femur configuration (Construct B), load sharing differences between the implant and the femur were computed from FE data as % difference = (implant peak strain - femur peak strain)/femur peak strain × 100 and % difference = (implant average stress - femur average stress)/femur average stress × 100, whereas the differences in implant peak stresses from FE were calculated as % difference = (Monoblock - Modular)/Modular × 100.

## Results

### FE results for implant-femur configuration

**Figure 5:**
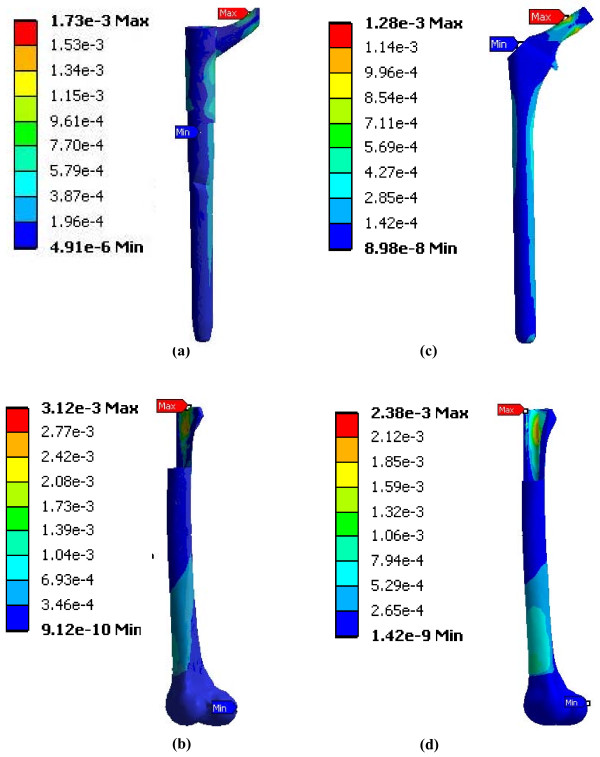
**FE model strain distributions of hip prostheses virtually implanted in FE models of a femur (Construct B)**. (a) Modular hip stem, (b) femur into which the Modular hip stem was inserted, (c) Monoblock hip stem, (d) femur into which the Monoblock hip stem was inserted. Strains shown are equivalent elastic strain (Von Mises) in mm/mm units. Results are for 2000 N axial load. Posterior view is shown.

**Figure 6:**
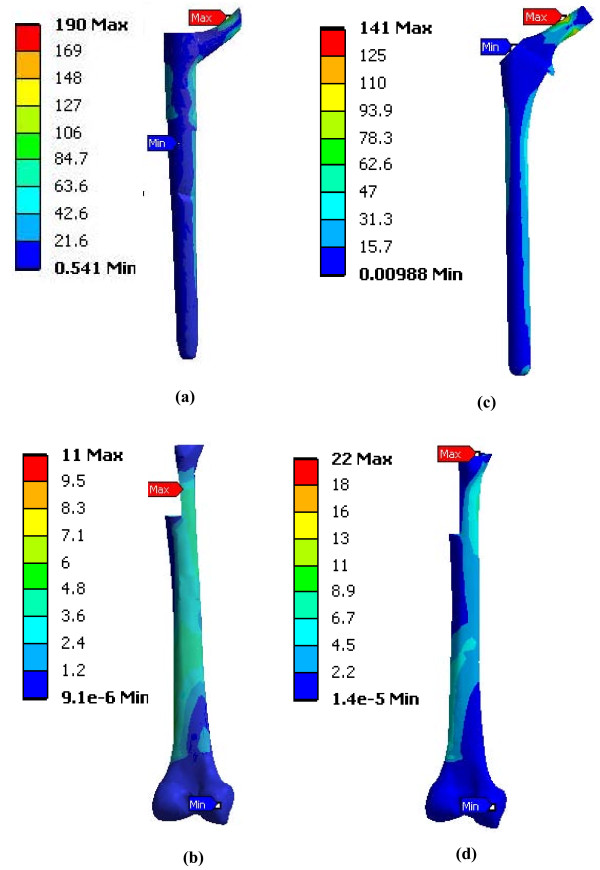
**FE model stress distributions of hip prostheses virtually implanted in FE models of a femur (Construct B)**. (a) Modular hip stem, (b) femur into which the Modular hip stem was inserted, (c) Monoblock hip stem, (d) femur into which the Monoblock hip stem was inserted. Stresses shown are equivalent elastic stresses (Von Mises) in MPa units. Results are for 2000 N axial load. Posterior view is shown.

**Figure 7:**
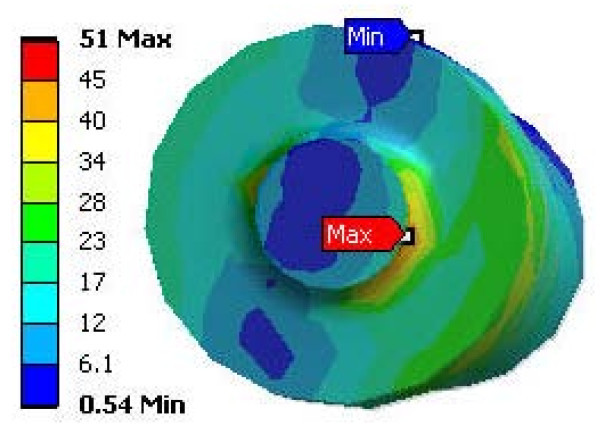
**Stress map of the Modular hip stem at the modular junction**. Under 2000 N axial load, a stress concentration is present at the modular junction. Stresses shown are equivalent elastic stresses (Von Mises) in MPa units.

The strain and stress distribution maps at 2000 N are shown for the implant-femur Construct B (Figures 5 and 6). Peak implant strains exceeded those of the host femur by 38% (Modular) and 39% (Monoblock). Peak implant stresses were situated in the neck region, particularly for the Modular on the stem at its body-stem junction (Figure 7). The Modular peak stresses exceeded that of the Monoblock implant by 34%. Average implant stress, excluding the peaks, ranged from 0 to 85 MPa (Modular) and 0 to 75 MPa (Monoblock). Both implants carried the majority (Modular, 76%; Monoblock, 66%) of the 2000 N axial force relative to the femur.

### Experimental results for implant alone

For Construct A (implant alone), stiffness values for the hip implants were 2476 N/mm (Modular) and 553 N/mm (Monoblock), indicating a 4.5 times difference between them. The linearity of the force-deflection data (Modular, R^2 ^= 0.968; Monoblock, R^2 ^= 0.997) showed that the specimens remained within the linear elastic region, incurring no permanent deformation during mechanical stiffness tests. Strain distributions obtained from experiments on the hip prostheses are shown (Table 1). By dividing strain values of corresponding Locations 1 to 8 at 2000 N by strain values at 700 N, the average strain ratios of 3.3 (Modular) and 3.2 (Monoblock) were obtained. These ratios were close to the ratio of the axial loads themselves (= 2000 N/700 N = 2.9). By dividing strain values of corresponding Locations 1 to 8 for the Monoblock by the strain values for the Modular hip implant, the average strain ratios of 3.1 (at 700 N) and 2.9 (at 2000 N) were obtained. This indicates that the Modular prosthesis was about 3 times stiffer than the Monoblock.

**Table 1:** Strains for hip implant alone (Construct A) for validating the FE model.

Side	Location	Modular (EXP)		Modular (FE)		Monoblock (EXP)		Monoblock (FE)	
		
		700 N	2000 N	700 N	2000 N	700 N	2000 N	700 N	2000 N
**Medial**	**1**	125	456	127	453	251	696	264	682
	
	**2**	144	523	157	524	259	789	249	756
	
	**3**	126	400	126	409	924	2966	482	1390
	
	**4**	190	543	200	570	894	2927	471	1319

**Lateral**	**5**	76	288	77	276	274	780	277	781
	
	**6**	80	274	82	268	188	677	172	673
	
	**7**	119	349	128	361	947	3077	392	1288
	
	**8**	290	767	298	776	821	2730	389	1221

Medial strains are compressive. Lateral strains are tensile. EXP = experimental values. FE = finite element analysis values.

### Validating the FE model using experimental results

For Construct A, the percentage difference between the FE model and experimental strain at Locations 1 to 8 were calculated as described earlier. For the Modular hip implant data (Table 1), the average differences for Locations 1 to 8 at axial loads of 700 and 2000 N, respectively, were 3.8 ± 3.2% (range, 0-9.0%) and 2.4 ± 1.7% (range, 0.2-4.9%). For the Monoblock hip implant data (Table 1), the average differences for Locations 1 to 8 at axial loads of 700 and 2000 N, respectively, were 28.1 ± 25.4% (range, 1.1-58.6%) and 28.6 ± 28.7% (range, 0.1-58.1%), with an overall average difference of 28.3 ± 26.2%. However, the Monoblock FE model was much more predictive for proximal Locations 1, 2, 5, and 6 (average % difference = 3.2 ± 2.8%), rather than for distal Locations 3, 4, 7, and 8 (average % difference = 53.4 ± 4.2%).

**Figure 8:**
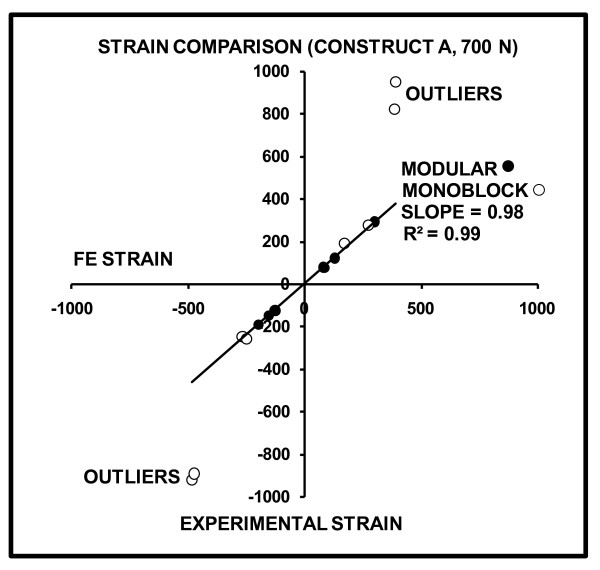
**FE-predicted versus experimentally-measured strains for Construct A at 700 N**. Positive values indicate tension, while negative values indicate compression. The slope and linearity coefficients (R^2^) of the line-of-best-fit for all Modular and proximal Monoblock strain gages are given. Perfect agreement between FE and experiments would yield slope = 1 and R^2 ^= 1. Outliers are gage Locations 3, 4, 7, and 8 on the distal portion of the Monoblock stem which demonstrated exaggerated experimental strain due to femoral ball movement in the medial direction under the load application plate.

**Figure 9:**
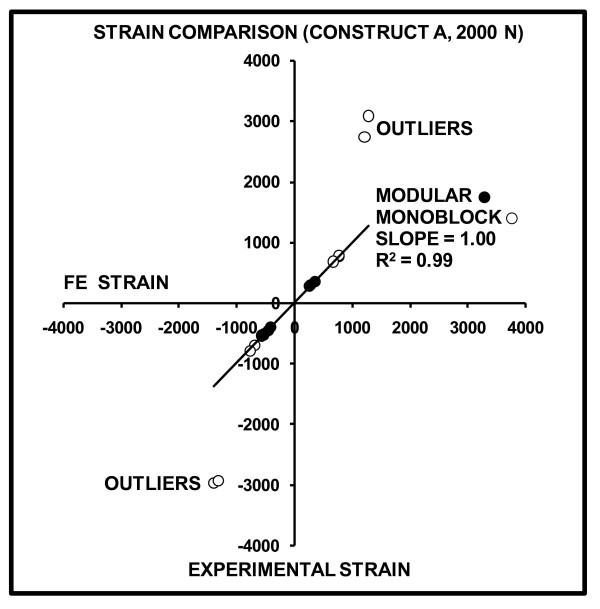
**FE-predicted versus experimentally-measured strains for Construct A at 2000 N**. Positive values indicate tension, while negative values indicate compression. The slope and linearity coefficients (R^2^) of the line-of-best-fit for all Modular and proximal Monoblock strain gages are given. Perfect agreement between FE and experiments would yield slope = 1 and R^2 ^= 1. Outliers are gage Locations 3, 4, 7, and 8 on the distal portion of the Monoblock stem which demonstrated exaggerated experimental strain due to femoral ball movement in the medial direction under the load application plate.

FE-predicted versus experimentally-measured strains for Construct A at 700 and 2000 N are graphically shown (Figures 8 and 9). The slopes and linearity coefficients (R^2^) of the lines-of-best-fit for all Modular and proximal Monoblock experimental strain values showed nearly perfect agreement with FE analysis, since results were close to the ideal values of slope = 1 and R^2 ^= 1. Poor agreement of "outliers" for the Monoblock stem was caused by the visually observed (but unmeasured) medial slippage of the femoral ball under the load application plate that resulted in exaggerated experimental strains. This medial motion of the femoral head was not considered in the FE model.

## Discussion

### FE model validation using experiments

The present three-dimensional FE model was validated experimentally for the hip implant alone (Construct A) at loads of 700 and 2000 N, showing reasonable agreement (Table 1). The Modular implant showed an overall average difference for all locations of 3.0% between FE and experimental values. The Monoblock device showed an average difference of 3.2% for the proximal Locations 1, 2, 5, and 6; however, the average difference was 53.4% for the distal Locations 3, 4, 7, and 8. This can be attributed to the medial slip of the femoral head under the load application plate that was visually observed (but not measured) during experiments, since motion was not constrained in this direction, causing exaggerated distal strains in the Monoblock device. This was not replicated in the FE model. In addition, it is likely that the lower stiffness Monoblock stem underwent medial slip more so than the higher stiffness Modular stem.

### Modular versus Monoblock hip implants

For Construct A (Table 1), FE and experimental strains showed that the Modular implant was about 3 times mechanically stiffer than the Monoblock, and the stiffness measurements showed the Modular device to be 4.5 times stiffer. This was most likely due to the difference in geometry of the devices with respect to their cross-sectional diameters in the transverse plane for the distal two-thirds of their lengths, namely, Modular (range, 16.5 to 20.2 mm) and Monoblock (range, 15.4 to 16.3 mm) (Figure 1). The material properties of the stems were virtually identical, since they were both made from titanium-based alloys. In the FE model, in fact, both implants had identical material properties assigned to them. Higher implant stiffness may create an implant-femur construct with an increased stiffness that provides enhanced mechanical stability in the immediate post-operative situation, but it may create eventual femur stress shielding and bone loss [23]. Conversely, lower device stiffness may result in increased load transfer to the host femur, subsequently stimulating bone ingrowth around the implant, minimizing bone loss due to resorption, and improving mechanical stability in the long-term [23-27].

For Construct B (Figure 6a and 6c), FE results showed that there was better overall load transfer to the whole femur and, thus, less load absorption by the Monoblock stem itself (0-75 MPa, excluding peaks) compared to the Modular implant (0-85 MPa, excluding peaks). Therefore, the Monoblock prosthesis might facilitate greater load transfer to the femur, potentially promoting increased bone ingrowth around the implant and reducing bone remodeling and resorption [23-27]. Conversely, the Modular device carried a greater amount of load, making it is less conducive to biomimetic performance in the patient femur. Moreover, the FE results identified a stress concentration on the stem at the modular junction of the Modular implant (Figure 7). This could make it susceptible to fracture clinically, in which the load levels may be elevated compared to that currently used, especially during higher impact activities which patients might choose to perform [21].

### Comparison of strains to prior studies

No earlier studies have examined the mechanical characteristics of the present Modular and Monoblock implants. However, comparison of current results to prior experiments on primary hip prostheses may be instructive. Waide and colleagues [28] found that experimental compressive microstrains on the medial side peaked at 756 for Muller-Curved and 765 for Lubinus SPII hip prostheses cemented into synthetic femurs. Akay and Aslan [20] measured experimental peak microstrains for hip implants cemented into synthetic femurs, held in 20 deg of adduction, and compressed with a 3000 N axial force. They obtained microstrains of almost 4000 (medial) and 2500 (lateral) for a carbon-based composite implant and about 1200 (medial) and 900 (lateral) for a titanium alloy prosthesis. These are similar to experimental strains achieved at present for Construct A (Table 1) and for FE analysis strains for Construct B (Figure 5) at 2000 N. Even so, proper inter-study comparison is difficult because of the variety of implant materials, implant geometries, and experimental conditions used in the literature, resulting in a broad range of strain levels achieved on implant surfaces.

### Clinical implications

Prior clinical results showed no problems at the Modular junction, though follow-up was limited to 2 and 5 years [10,11]. Other clinical results showed that the junction is a site for potential implant fracture, often being associated with proximal bone loss causing implant loosening and increased surface stresses [29,30]. This agrees with maximum stem strains noted presently for the Modular device at its modular junction (i.e., at the proximal and distal interface) (Figure 5). This may lead to premature implant failure, especially at higher loads than those examined at present [21]. The Modular device transferred higher loads to the distal half of the femur bone itself (0-7.5 MPa, including peaks) than the Monoblock device was able to achieve (0-6.9 MPa, including peaks) (Figure 6b and 6d). As confirmed clinically [31,32], increased load transfer to the distal femur could cause bone densification around the distal implant tip and possible femur fracture. Conversely, the single-piece Monoblock implant had limited strain concentrations towards the proximal neck with increasing strains cascading down towards its distal end. This was punctuated by a peak stress at the distal-most medial point near the cement chamber. While this did not replicate a clinical scenario, it did suggest that the Monoblock device offered more anatomical loading than the Modular implant. Accordingly, the Monoblock prosthesis may have replicated the natural femur better, promoting superior load transfer.

### Limitations

Firstly, only axial compression tests were assessed. The hip device would clinically be exposed to dynamic forces exceeding the 700 and 2000 N used presently due to muscle action and activities of daily living [21,33]. Given identical host bone quality, muscle activity, and soft tissue constraint, each of which have an influence on the dynamic forces and moments experienced by the bone-implant construct, it is anticipated that the relative performance reported of the Modular versus the Monoblock hip prostheses would be similar in a "real world" clinical scenario. However, the absolute values of stress and strain obtained at present may not be reflective of in vivo results. A direct biomechanical comparison between the stems in vivo would be required to demonstrate these postulations conclusively.

Secondly, the FE model used to assess Construct B assumed that the synthetic femur had linear isotropic mechanical properties. This simplified the analysis significantly. However, nonlinearity, anisotropy, and viscoelasticity may influence bulk mechanical behavior of the bones. Even so, comparison of FE analysis, synthetic femurs, and human cadaveric femurs previously performed by some of the authors [14] showed that linear behavior is a good approximation of the femurs in axial compression and torsion. Moreover, these assumptions have been used in the literature previously for synthetic femur FE modeling [14-16,26].

Thirdly, FE analysis had limited success in assessing the experimental performance at the distal end of the Monoblock device for Construct A. This was likely due to the visual observation that the implant shifted medially under the load application plate, since motion was not constrained in that direction, thereby exaggerating distal strains. This was not replicated in the FE analysis. Moreover, it is possible that the lower stiffness Monoblock stem underwent such medial slip more so than the higher stiffness Modular stem. Nonetheless, the FE model was able to replicate all Modular strains and proximal Monoblock strains to within about 3% of experimental data.

Fourthly, the FE model mimicked long-term bone ongrowth around the stem and mechanical stability by employing bonded contact at all implant-bone, implant-cement, and implant-implant interfaces, i.e., bone absorption/apposition was neglected. However, in actuality, ongoing bone remodeling over the life span of the implant will alter implant-femur biomechanics. The current FE model, in effect, only assessed the biomechanical situation at one time point during the service life of the hip stem.

Fifthly, equivalent Von Mises strains from FE analysis were used for comparison to experimental values from uniaxial linear strain gages. It could be suggested that a more appropriate technique would have been to compare only FE principal strains with linear strain gage results. However, it must be noted that the current approach has been used by some of the present authors and others in prior studies of bone-implant biomechanics [15,16]. Moreover, the strain gages are physically large, so that they actually detect the average surface strain over a given area, which is different than the principal strain at a specific point. Furthermore, comparing equivalent Von Mises strains from FE with experimental values may have been especially appropriate for slightly curved surfaces, i.e. Locations 1, 2, and 5, since the slightly curved uniaxial strain gages were essentially providing an average reading of strain along the length of an arc. Ideally, however, rosette-type strain gage setups may be able to provide more exact experimental readings for comparison to Von Mises strains from FE analysis, especially when circumferential strains are also of interest [19,20].

Finally, this study combined into an FE implant-femur model (Construct B) separate FE models for the hip implants alone presently and the synthetic femur from prior investigations [14-16]. Although individual implant and femur FE models have been validated, future work may ideally include experimentally testing an actual implant-femur construct. The reason the present authors performed separate experimental validations of the FE models for the stems alone and the femur alone was a pragmatic one. Specifically, physically inserting hip stems into the synthetic femur canal would have made strain gage measurements along the surface of the hip stem itself extremely difficult experimentally because of the very fragile nature of strain gages (i.e. they easily peel or shear off) and the confined space inside the canal (i.e. there is little room to accommodate strain gage wiring).

In spite of these drawbacks, the main intention of this study was only to provide a preliminary assessment of the biomechanics of the Modular and Monoblock stems upon which more comprehensive future work may be built.

## Conclusions

This is the first report of the biomechanical characteristics of these particular two-piece Modular and single-piece Monoblock revision hip implants. The FE model of the hip implant alone (Construct A) was predictive of experimental strains at all locations on the Modular device, but only for the proximal portion in the Monoblock device. Significant differences in FE strains of the two prostheses implanted in an FE femur model (Construct B) signified a greater amount of stress absorption by the Modular implant than in the Monoblock, suggesting that the host femur may carry more of the load when the Monoblock device is employed. A stress concentration on the Modular implant occurred at the modular junction, which may be susceptible to failure; however, no comparable potential failure points were identified on the Monoblock prosthesis. Experiments on Construct A showed that the Modular device was 3 to 4.5 times mechanically stiffer than the Monoblock.

## List of Abbreviations

Construct A: implant alone; Construct B: femur-implant construct; E: Young's Modulus; EXP: experimental values; FE: finite element; R^2^: Pearson linear correlation coefficient; ν: Poisson's ratio.

## Competing interests

The authors declare that they have no competing interests.

## Authors' contributions

HB, RZ, MP, and PZ were involved in initial study design, femur acquisition, and implant acquisition. HB, MM, and MP were involved in FE modeling and analysis. RZ and SS were involved in specimen preparation, mechanical testing, and data analysis. HB, RZ, and MM did the literature search, manuscript writing, figure preparation, and data analysis. MP and PZ did extensive re-reading of the manuscript. PZ and EHS were involved in general supervision and consultation of the project regarding clinical relevance. All authors approve of this final manuscript version.

## References

[B1] Hall MJ Owings MF 2000 National Hospital Discharge Survey Adv Data 2002 329 1 18 full_text 12664934

[B2] Furey MJ Peterson DR, Bronzino JD Joint Lubrication Biomechanics: Principles and Application 2008 Boca Raton, USA: CRC Press 4-1 4-25

[B3] Wheeless' Textbook on Orthopaedics http://www.wheelessonline.com

[B4] Cameron HU Modular junctions Orthopedics Sep 2005 28 9 Suppl S1057 1058 10.3928/0147-7447-20050902-11 16190036

[B5] Fink B Grossmann A Schubring S Schulz MS Fuerst M Short-term results of hip revisions with a curved cementless modular implant in association with the surgical approach Arch Orthop Trauma Surg 2009 129 1 65 73 10.1007/s00402-008-0617-7 18389264

[B6] Gacon G Philippe MP Ray A Hummer J Hourlier H Dambreville A [Metaphyseal and diaphyseal modular femoral implants implanted without cement] [Article in French] Rev Chir Orthop Reparatrice Appar Mot 2001 87 4 331 339 11431628

[B7] Garcia-Rey E Muñoz T Montejo J Martinez J Results of a Hydroxyapatite-Coated Modular Femoral Stem in Primary Total Hip Arthroplasty. A Minimum 5-Year Follow-Up J Arthroplasty 2008 23 8 1132 1139 10.1016/j.arth.2007.10.012 18534471

[B8] Lombardi AV Jr Berend KR Mallory TH Adams JB Modular calcar replacement prosthesis with strengthened taper junction in total hip arthroplasty Surg Technol Int 2007 16 206 209 17429790

[B9] Middleton RG Howie DW Costi K Sharpe P Effects of design changes on cemented tapered femoral implant fixation Clin Orthop Relat Res 1998 355 47 56 10.1097/00003086-199810000-00006 9917590

[B10] Cherubino P Surace MF Zatti G [Implant revision: special implant versus primary device.] [Article in English, Italian] Chir Organi Mov 2003 88 3 281 284 15146945

[B11] Kang MN Huddleston JI Hwang K Imrie S Goodman SB Early outcome of a modular femoral component in revision total hip arthroplasty J Arthroplasty 2008 23 2 220 225 10.1016/j.arth.2007.03.006 18280416

[B12] Zimmer http://www.zimmer.com

[B13] Stryker http://www.stryker.com

[B14] Papini M Zdero R Schemitsch EH Zalzal P The biomechanics of human femurs in axial and torsional loading: comparison of finite element analysis, human cadaveric femurs, and synthetic femurs J Biomech Eng 2007 129 1 12 19 10.1115/1.2401178 17227093

[B15] Cheung G Zalzal P Bhandari M Spelt JK Papini M Finite element analysis of a femoral retrograde intramedullary nail subject to gait loading Med Eng Phys 2004 26 2 93 108 10.1016/j.medengphy.2003.10.006 15036177

[B16] Bougherara H Zdero R Miric M Shah S Hardisty M Zalzal P Schemitsch EH The Biomechanics of the T2 Femoral Nailing System: A Comparison of Synthetic Femurs with Finite Element Analysis Proc Instn Mech Engrs, Part H: J Engineering in Medicine 2009 223 H3 303 314 10.1243/09544119JEIM501 19405436

[B17] Talbot M Zdero R Schemitsch EH Cyclic Loading of Periprosthetic Fracture Fixation Constructs J Trauma 2008 64 5 1308 1312 10.1097/TA.0b013e31811ea244 18469655

[B18] Zdero R Walker R Waddell JP Schemitsch EH Biomechanical Evaluation of Periprosthetic Femoral Fracture Fixation J Bone Joint Surg Am 2008 90 5 1068 1077 10.2106/JBJS.F.01561 18451400

[B19] Decking R Puhl W Simon U Claes LE Changes in strain distribution of loaded proximal femora caused by different types of cementless femoral implants Clin Biomech 2006 21 495 501 10.1016/j.clinbiomech.2005.12.011 16457913

[B20] Akay M Aslan N Numerical and experimental stress analysis of a polymeric composite hip joint prosthesis J Biomed Mater Res 1996 31 167 182 10.1002/(SICI)1097-4636(199606)31:2<167::AID-JBM3>3.0.CO;2-L 8731205

[B21] Bergmann G Graichen F Rohlmann A Hip joint loading during walking and running, measure in two patients J Biomech 1993 26 8 969 990 10.1016/0021-9290(93)90058-M 8349721

[B22] Pedersen DR Brand RA Davy DT Pelvic muscle and acetabular contact forces during gait J Biomech 1997 30 9 959 965 10.1016/S0021-9290(97)00041-9 9302620

[B23] Sumner DR Turner TM Igloria R Urban RM Galante JO Functional adaptation and ingrowth of bone vary as a function of hip implant stiffness J Biomech 1998 31 909 917 10.1016/S0021-9290(98)00096-7 9840756

[B24] Jacobs JJ Sumner DR Galante JO Mechanisms of bone loss associated with total hip replacement Orthop Clin North Am 1993 24 583 590 8414423

[B25] Nishii T Sugano N Masuhara K Shibuya T Ochi T Tamura S Longitudinal evaluation of time related bone remodeling after cementless total hip arthroplasty Clin Orthop Relat Res 1997 339 121 131 10.1097/00003086-199706000-00017 9186210

[B26] Speirs AD Heller MO Taylor WR Duda GN Perka C Influence of changes in stem positioning on femoral loading after THR using a short-stemmed hip implant Clin Biomech 2007 22 431 439 10.1016/j.clinbiomech.2006.12.003 17275151

[B27] Van Rietbergen B Huiskes R Weinans H Sumner DR Turner TM Galante JO The mechanism of bone remodeling and resorption around press-fitted stems J Biomech 1993 26 369 382 10.1016/0021-9290(93)90001-U 8478342

[B28] Waide V Cristofolini L Stolk J Verdonschot N Toni A Experimental investigation of bone remodeling using composite femurs Clin Biomech 2003 18 523 536 10.1016/S0268-0033(03)00072-X 12828902

[B29] Pierson JL Crowninshield RD Earles DR Fatigue fracture of a modular revision femoral component: a report of forty cases Am Acad Orthop Surg, Annual Meeting, Washington, DC, 23-27 February, 2005

[B30] Crowninshield RD Maloney WJ Wentz DH Levine DL The role of proximal femoral support in stress development within hip prostheses Clin Orthop Rel Res 2004 420 176 180 10.1097/00003086-200403000-00024 15057094

[B31] Gupta S New AMR Taylor M Bone remodelling inside a cemented resurfaced femoral head Clin Biomech 2006 21 6 594 602 10.1016/j.clinbiomech.2006.01.010 16542761

[B32] Theis JC Ball C Medium-term results of cementless hydroxyapatite-coated primary total hip arthroplasty: a clinical and radiological review J Orthop Surg (Hong Kong) 2003 11 2 159 165 14676341 10.1177/230949900301100210

[B33] Duda GN Heller M Albinger J Schulz O Schneider E Claes L Influence of muscle forces on femoral strain distribution J Biomech 1998 31 841 846 10.1016/S0021-9290(98)00080-3 9802785

